# Covid-19 and Mental Health: Could Visual Art Exposure Help?

**DOI:** 10.3389/fpsyg.2021.650314

**Published:** 2021-04-30

**Authors:** Laura M. H. Gallo, Vincent Giampietro, Patricia A. Zunszain, Kai Syng Tan

**Affiliations:** ^1^Department of Psychological Medicine, Institute of Psychiatry, Psychology and Neuroscience, King’s College London, London, United Kingdom; ^2^Department of Neuroimaging, Institute of Psychiatry, Psychology and Neuroscience, King’s College London, London, United Kingdom; ^3^Manchester School of Art, Manchester Metropolitan University, Manchester, United Kingdom

**Keywords:** neuroesthetics, reward pathways, mental resilience, fMRI, art

## Abstract

A worldwidemental health crisis is expected, as millions worldwide fear death and disease while being forced into repeated isolation. Thus, there is a need for new proactive approaches to improve mental resilience and prevent mental health conditions. Since the 1990s, art has emerged as an alternative mental health therapy in the United States and Europe, becoming part of the social care agenda. This article focuses on how visual esthetic experiences can create similar patterns of neuronal activity as those observed when the reward system is activated. The activation of the reward structures could have a stress buffering effect, given the interdependence observed between the reward and stress systems. Therefore, could visual esthetic experiences stimulate mental resilience? And if this were the case, could art-based interventions be offered for mental health in the context of COVID-19 and beyond?

## Introduction

Isolation, fear, and financial/occupational instability created by the current COVID-19 situation are expected to generate an upsurge in mental illnesses globally (Rajkumar, 2020; Torales et al., 2020). Shortage of mental health workforce and the financial resources needed for traditional interventions are limiting factors to cope effectively with a potential global mental health crisis. United Kingdom research already shows an increase in levels of anxiety, depression, and stress due to current financial challenges (Türközer and Öngür, 2020). This reinforces the urgent need to discover new complementary interventions to help improve low mood and alleviate mental health risks (Holmes et al., 2020).

Just like cognitive behavioral therapy went online to reach more people, equal efforts should be undertaken to deliver art interventions remotely, reaping the benefits of its positive cognitive effects. Here, we discuss how reward brain activation following visual art exposure could promote stress-buffering effects, based on the interdependence observed between the rewards pathway and the sympathetic nervous system. These observations would help to show how visual esthetic experiences could stimulate mental resilience.

## Art on Prescription

Art interventions have been linked to healing and recovery, influencing mental, somatic, and psychological conditions (Samaritter, 2018; Mastandrea et al., 2019a). This has motivated scientists to study the sensory-emotional values that art can elicit. The first United Kingdom program of arts on prescription was designed in 1994 to help recovery of mild and moderate depression (Bungay and Clift, 2010). Visual art interventions, including painting (Bar-Sela et al., 2007) and collage making (Forzoni et al., 2010) have also been considered helpful by patients suffering from depression or fatigue during chemotherapy treatment.

Assessing the effectiveness of these interventions has been challenging due to a lack of scientific rigor of the rating instruments (Betts, 2006). Nevertheless, a qualitative study of 102 art and mental health projects concluded that art participation enhanced levels of empowerment and was associated to improvements in participants’ mental health (Spandler et al., 2007).

Despite a large cohort study suggesting that individuals experiencing depression or anxiety could struggle to engage in art activities (Fancourt and Finn, 2019; Fancourt et al., 2020), a survey conducted by the United Kingdom charity Mind showed that 70% of service users placed art-based therapies among their top three treatment choices (Parsons et al., 2019). Based on these observations, Parsons et al. (2019) are trialing a combination of mainstream talking therapies and art-based interventions. This mixed approach should help to overcome the limitations of each treatment providing an alternative way to express feelings that may be otherwise difficult or uncomfortable to admit and discuss. Art interventions could be considered as an additional alternative for integrative medicine, becoming a supportive tool in a similar way to mindfulness (Garland et al., 2009; Moss, 2018).

Additionally, a connection between increased art making and neurodegeneration has been reported in Parkinson’s disease patients after they started taking dopamine-boosting medication, reinforcing the idea of a relationship between art and the reward system (Canesi et al., 2016). Drawing on these developments, health policy makers are increasingly adopting art-based interventions. Systematic reviews have been conducted using studies from in the United Kingdom, the United States, Netherlands, Sweden, Israel, and France to determine their clinical effectiveness, concluding that art-based therapies are acceptable cost-effective treatments (Reynolds et al., 2000; Leurent et al., 2014; Uttley et al., 2015). Therefore, studying the underlying neural mechanisms of visual art stimulation, which is one focus of the cognitive field of Neuroesthetics, could help us to further understand its benefits.

## Neuroesthetics: Visual Esthetic Experience

Neuroesthetics studies (Brown et al., 2011; Skov, 2019) show how the brain reacts to most forms of art (paintings, music, dance, etc.). In the case that interests us here, i.e., visual art, the affective meanings of color, line or shape (effortless unconscious “bottom up” processes) have been found to activate reward system structures (Pelowski et al., 2017). When the art object was associated with a positive social construct (effortful conscious “top down” process), the added “interpretation” further enhanced the esthetic experience (Barry, 2006). Numerous fMRI studies have identified the brain regions associated with perceptual, cognitive, emotional, and reward processing when assessing the beauty of images (Brown et al., 2011; Chatterjee, 2011; Nadal and Skov, 2013; Skov, 2019).

## Reward Areas Activated by Art and Their Connection to the Stress Responsive System

Viewing paintings generate remarkably similar patterns of brain activity as other pleasurable stimuli, like food, sex, or addictive drugs (Berridge and Kringelbach, 2015). In addition to the basic sensory and social pleasures rewards shared with most animals, humans also have high-order pleasure rewards (Berridge and Kringelbach, 2008). In order to investigate if brain activity could differ between these reward types, Sescousse et al. (2013) compared the brain responses associated with monetary, erotic, and food reward stimuli. They observed that monetary rewards showed higher activation in the orbitofrontal cortex (OFC), compared to the other reward types, indicating that abstract secondary rewards could be associated with more evolved brain regions, which was also the case with art rewards. According to Levy and Glimcher (2012), dissimilar type of rewards can be considered equally desirable for the same individual showing similar OFC activation, supporting the neural common currency hypothesis.

Ultimately, all reward types were associated with a consistent activity increase in the OFC, amygdala, and ventral striatum/nucleus accumbens (McClure et al., 2004). These are the same brain structures that have been shown to be activated during esthetic experiences (Boccia et al., 2016).

**Figure 1:**
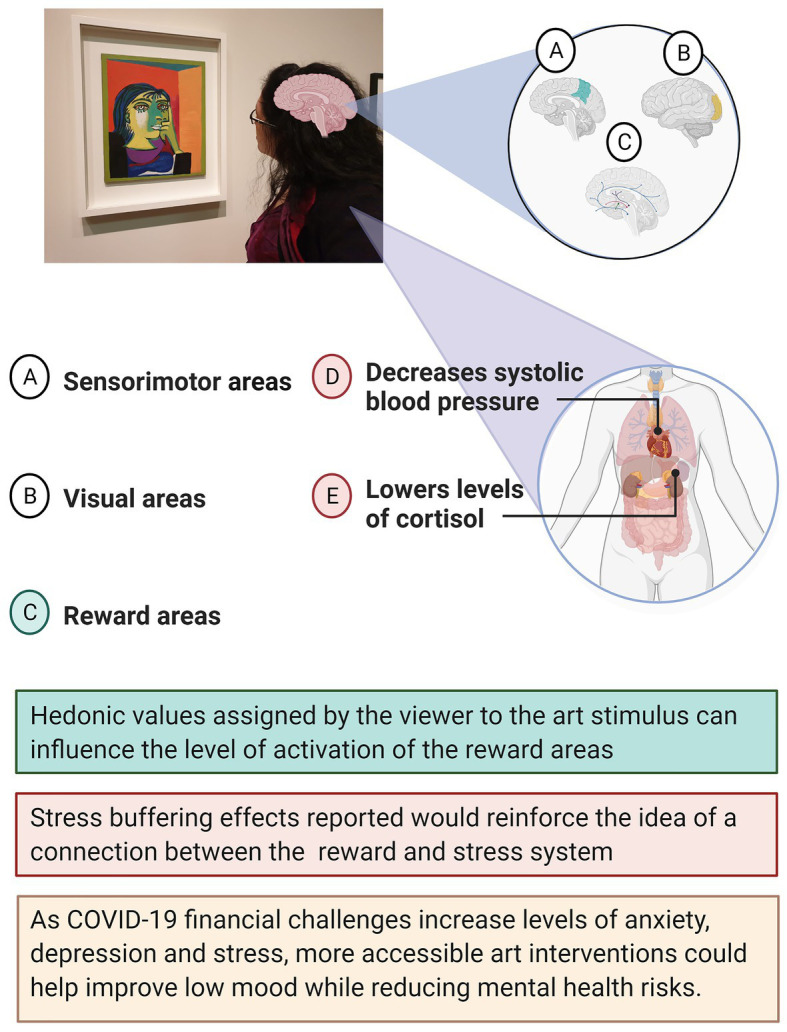
Schematic diagram for neural activation and stress buffering physical effects of visual art exposure.

Figure 1 illustrates how brain activation is not limited to the visual and sensorimotor areas but can also engage the reward network through a positive correlation between assigned hedonic value and activation levels in reward areas (Vartanian and Goel, 2004). A meta-analysis comprised of 330 participants conducted by Vartanian and Skov (2014) found that viewing paintings activated the brain’s emotion and reward systems in addition to the expected visual cortex. A limitation of these findings could be the prevalence of small sample sizes (mean 22 ± 4.49). The benefits of art have also been measured through biological parameters of the central nervous system activity. For example, visits to art museums have reported to lower levels of salivary cortisol and self-reported measure of stress (Clow and Fredhoi, 2006) and to decrease systolic blood pressure (Mastandrea et al., 2019b). Taking into consideration the many benefits of art interventions, making them more accessible could be part of the wider strategy of managing mental health risk and treatment. The reward pathway activation observed as a consequence of visual art exposure can be beneficial as reward activation has shown to modulate survival behaviors by reducing stress levels. Even brief exposure to reward stimuli (such as erotic images) can decrease cortisol reactivity, improving cognitive performance on social stress tasks (Creswell et al., 2013).

It is important to note that reward system projections have been linked to regulation of stress response and decreased hypothalamo-pituitary-adrenal response (HPA; Diorio et al., 1993). Dopaminergic neurons of the ventral tegmental area (VTA), which modulates stress vulnerability (Gillies et al., 2014), have dopaminergic connections to the same reward system structures (amygdala, ventral striatum, and OFC) that have been found to become activated by visual art stimuli, which we discuss in more detail below.

### The Orbitofrontal Cortex

The OFC sits within the neocortex and has been associated to emotions, decision making, and rewards processing (Wallis, 2007; Rolls and Grabenhorst, 2008; Bray et al., 2010). Several studies have suggested that the OFC is involved in making esthetic judgments and in conferring hedonic values (Kringelbach, 2005; Kirk et al., 2009; Huang et al., 2011; Ishizu and Zeki, 2011, 2013; Lacey et al., 2011; Vessel et al., 2012). Furthermore, contextual information might influence the activity observed, as Kirk et al. (2009) found when comparing activation between images labeled as gallery or computer-generated. Gallery labeled images were associated with a significantly higher OFC activation compared to computer labeled images, despite using the same artworks and randomly labeling the images. Nevertheless, some studies with similar designs have failed to observe such OFC activation (Vartanian and Goel, 2004; Cupchik et al., 2009). Lacey et al. (2011) argued that cortical areas adjacent to sinuses, like the OFC, can be susceptible to signal drop-out (Li et al., 1996) which may, in part, explain this inconsistency.

The OFC also makes a significant contribution to the regulation of autonomic stress responses, providing inputs to the hypothalamus (Hostinar et al., 2014). Furthermore, abnormal stress response has been associated with OFC hypoactivation (Holsen et al., 2012). Future studies could investigate if pleasant esthetic experiences help improve this dysregulation.

### The Striatum

The striatum is composed of three nuclei: ventral striatum (VS), caudate, and putamen (Báez-Mendoza and Schultz, 2013). This brain structure, involved in reward-related processing (Delgado, 2007), was also found to be activated during esthetic experiences. Studies using western art images have reported activation in the VS (Lacey et al., 2011; Vessel et al., 2012), the caudate nucleus (Vartanian and Goel, 2004; Ishizu and Zeki, 2011), and the putamen (Ishizu and Zeki, 2013). Lacey et al. (2011) explored if VS activation could be influenced by artistic status. They paired 50 paintings with “ordinary pictures” that resembled the content and layout of the artwork, finding significantly stronger activation of the VS for art images compared to non-art ones. Likewise, the right caudate nucleus has demonstrated a significant positive correlation between activation level and art image preference. Decreased activation matched lower art ratings, becoming more evident when minimal activation indicated very low rated art (Vartanian and Goel, 2004). Ishizu and Zeki (2013) compared perceptual judgment (brightness) and affective judgment (esthetic) to study if both were recruiting the same brain areas. Participants had to make either an esthetic judgment (“which one is more beautiful?”) or a brightness judgment (“which one is brighter?”). Only the esthetic judgment condition showed activation of the basal ganglia, the putamen, and the globus pallidus. Moreover, it has been shown that changes in reinforcement events, such as having fulfilling relationships or feeling productive, lead to decreased energy and mood and can conduce to abnormal ventral striatum functional connectivity, which has been found to be a predictor of depressive disorder risk (Pan et al., 2017). Considering the rewarding aspect of esthetic experiences, art consumption could constitute a self-sustained reinforcement event to help with low mood and reduce the risk of abnormal functional connectivity.

### The Amygdala

Although the amygdala has been consistently associated with emotions, especially fear conditioning (Krabbe et al., 2018), it has also been shown to be associated with reward related learning. A rodent study has observed amygdala involvement in updating reward value (Chesworth and Corbit, 2017) and this brain area was also shown to be involved in experiences of pleasure or disgust when assessing facial attractiveness in humans (Winston et al., 2007). Signal drop-out due to magnetic inhomogeneity and low signal-to-noise ratio in this region might be a reason for which neuroesthetic studies do not consistently report activation in the amygdala (Boubela et al., 2015). Another explanation could be that the amygdala is implicated in esthetic or affective judgment (Wagar and Thagard, 2004), rather than in simply viewing the art. It is worth noting that the neuroesthetic studies reporting amygdala activation included such judgment tasks (Ishizu and Zeki, 2011, 2013; Jacobs et al., 2016) which were not included in those studies that did not report amygdala activation.

The amygdala has also been shown to be recruited in several tasks that require emotional decision-making, such as expression and perception of fear, appetitive processes, or social judgments, and it is part of the stress-response system (Drevets, 2003). Mood disorders, like major depression or bipolar disorder have been associated with elevated resting cerebral blood flow in this region, with downregulation depending on cognitive control areas, like the OFC, to reappraise contextual value (Dolan, 2007). In essence, the role of the amygdala in emotional regulation, which has been extensively studied, might explain its involvement during esthetic judgments, as subjective preferences are mostly based upon emotional responses.

## Discussion

As the COVID-19 variants threaten to extend the duration of this pandemic, the widespread emotional distress increases the risk for psychiatric illness. In light of this potential mental health crisis, which is even affecting the health workforce, new self-help tools to enhance mental resilience need to be identified and implemented. As explained above, there is enough evidence to suggest that art interventions can support mental health recovery, thanks to its emotionally charged rewarding nature.

Translational and human research has consistently shown that increased reward manipulations and rewarding environments promote the growth of stress resilience (Dutcher and Creswell, 2018). Much of this research has analyzed the stress buffering effects of primary rewards using food, drink, or sex (Abad et al., 1996; Christiansen et al., 2011; Creswell et al., 2013). However, there is also emerging evidence that visual art interventions can enhance functional connectivity related to psychological resilience (Bolwerk et al., 2014), can lower levels of salivary cortisol and of self-reported measure of stress (Clow and Fredhoi, 2006) and even decrease systolic blood pressure (Mastandrea et al., 2019b).

Examples of association between the reward and stress systems have been documented, such as in the animal and human findings on how psychological stress can affect eating behavior (Levine and Morley, 1981; Morley et al., 1983; Adam and Epel, 2007) or how environmental stressors can decrease reward-seeking behavior leading to anhedonia (Stanton et al., 2019). Stress responses regulated by the HPA system control body peripheral functions, like metabolism and immunity, while having an intense effect on the brain (Pariante and Lightman, 2008). For example, the hypothalamus has GABAergic connections to the reward system, connecting to the Ventral Striatum (Russo and Nestler, 2013), reinforcing the idea that reward and stress systems are linked. Resilient special forces soldiers revealed unique activation patterns during anticipation of reward (Vythilingam et al., 2009), while a large fMRI study suggested that resilience to adversity was correlated with increased dopaminergic activity in the VTA and hippocampus (Richter et al., 2019).

The close relationship between these systems could explain how art that stimulates our reward system (Nadal, 2013) can promote well-being, social inclusion and support mental health recovery and resilience. Art can help express complex feelings or sensations, avoiding verbal interpretations while allowing for diversion and emotional escape, as has been shown, even during extremely challenging situations, like imprisonment (Gussak, 2007). Given the psychological parallels that can be drawn between imprisonment and COVID-19 lockdown policies (Dhami et al., 2020), art could also, therefore, provide a coping mechanism to counter the negative consequences of this form of confinement.

On the basis of the evidence presented here, we recommend that resources can be allocated for new online art therapy treatment for psychological stress and low mood to improve the general population’s mental well-being and resilience, which is especially important in this time of crisis while the researchers are still assessing the psychological impact of the COVID-19 crisis.

## Author Contributions

This opinion article was conceived and processed into the manuscript by LG under supervision of VG, PZ, and KT. All authors critically appraised the intellectual content and structure of this manuscript and approved the final version.

### Conflict of Interest

The authors declare that the research was conducted in the absence of any commercial or financial relationships that could be construed as a potential conflict of interest.
